# Comparison of Healthcare Workers Transferring Patients Using Either Conventional Or Robotic Wheelchairs: Kinematic, Electromyographic, and Electrocardiographic Analyses

**DOI:** 10.1155/2016/5963432

**Published:** 2016-05-31

**Authors:** Hiromi Matsumoto, Masaru Ueki, Kazutake Uehara, Hisashi Noma, Nobuko Nozawa, Mari Osaki, Hiroshi Hagino

**Affiliations:** ^1^Rehabilitation Division, Tottori University Hospital, Nishi-cho 36-1, Yonago, Tottori 683-8504, Japan; ^2^Center for Promoting Next-Generation Highly Advanced Medicine, Tottori University Hospital, Nishi-cho 36-1, Yonago, Tottori 683-8504, Japan; ^3^Department of Data Science, The Institute of Statistical Mathematics, Midori-cho 10-3, Tachikawa, Tokyo 190-8562, Japan; ^4^School of Health Science, Faculty of Medicine, Tottori University, Nishi-cho 86, Yonago, Tottori 683-8503, Japan

## Abstract

*Objectives*. The aim of this study was to compare the musculoskeletal and physical strain on healthcare workers, by measuring range of motion (ROM), muscle activity, and heart rate (HR), during transfer of a simulated patient using either a robotic wheelchair (RWC) or a conventional wheelchair (CWC).* Methods*. The subjects were 10 females who had work experience in transferring patients and another female adult as the simulated patient to be transferred from bed to a RWC or a CWC. In both experimental conditions, ROM, muscle activity, and HR were assessed in the subjects using motion sensors, electromyography, and electrocardiograms.* Results*. Peak ROM of shoulder flexion during assistive transfer with the RWC was significantly lower than that with the CWC. Values for back muscle activity during transfer were lower with the RWC than with the CWC.* Conclusions*. The findings suggest that the RWC may decrease workplace injuries and lower back pain in healthcare workers.

## 1. Introduction

Musculoskeletal and physical strain caused by patient handling are prevalent among healthcare workers. In particular, musculoskeletal pain and lower back muscle injuries in nurses working in the geriatric setting are very high because of the significant number of patient transfers to wheelchairs that involve lifting [1–4]. It has been reported that physical therapists working in rehabilitation settings who perform 6–10 patients transfers per day are 2.4 times more likely to develop lower back injuries than therapists who do not perform transfers [5]. A previous study on occupational and physical therapists showed that transferring or lifting patients was associated with 26.6% of all injuries during work-related activities [6]. Moreover, a study on various nursing work activities showed that during the transfer of patients, the nurses' heart rate (HR) increased to approximately 125 beats/min (bpm) and they had higher levels of neuromuscular fatigue [4]. Therefore, the transfer of patients to wheelchairs produces increased burden on the musculoskeletal and cardiovascular systems through changes in joint range of motion (ROM), muscle activity, and HR. Therefore, there is a higher probability of musculoskeletal and physical strain in healthcare workers who transfer patients.

Several interventions have been reported to decrease musculoskeletal injuries in healthcare workers [7–9]. In fact, the implementation of safe patient handling and movement policies by the nursing profession has dramatically decreased work-related injuries and chronic pain [9]. Nurses who are more skilled in patient transfer increase the patients' perceptions of safety and comfort during transfers [8]. Some previous literature reported the efforts to decrease musculoskeletal injuries; however, strong evidence for the effectiveness of intervention is lacking.

On the other hand, following the adoption of no-lift policies, transfer robotic devices have emerged as tools that have the potential to prevent injuries in healthcare workers [10]. Robotic lift and powered devices may decrease both the patients' effort and the clinicians' physical burden. One tool emerging from these initiatives is a battery-powered sit-to-stand transfer device that safely lifts and lowers patients between the seated and standing positions [11, 12]. Transfer of patients with disabilities who are unable to contribute their own effort depends on powered robotic devices. However, the patients' physical activity or motivation may increase if the patients can transfer themselves using these assistance devices.

Recently, a new robotic wheel chair (RWC) has been developed which enables patients to be transferred directly in the sitting position using their own effort and the assistance of a healthcare worker [13]. The manual transfer of disabled patients from the bed to a conventional wheelchair (CWC) is demanding and involves complex movements. Patient-handling tasks involved with a CWC can be classified into 3 groups: lifting of the patient, repositioning or turning from the bed towards the direction of the wheelchair, and seating the patient safely in the chair. However, the RWC involves only 1 transfer step, which is that the assistant pushes the patient sitting on a bed forward in the same position to the seat of the RWC. Therefore, the RWC may decrease the complexity of transfer and decrease physical load during transfer for healthcare workers.

The purpose of this experimental study was to investigate the burden on healthcare workers by measuring ROM, muscle activity, and HR during transfer of a simulated patient using either the RWC or a CWC.

## 2. Methods

### 2.1. Participants

Ten females adults were recruited from an acute hospital and included 6 nurses and 4 rehabilitation therapists who had work experience in transferring patients (mean age: 32.2 ± 9.3 years; range: 23–47 years; body weight: 48.8 ± 4.7 kg; height: 157.2 ± 6.7 cm; BMI: 19.7 ± 1.3 kg/m^2^). Another female adult (age: 27 years; body weight: 49.0 kg; height: 153.0 cm; BMI: 20.9 kg/m^2^) participated in the study acting as the simulated patient who was transferred from bed to the wheelchairs. The simulation was assumed to be a right hemiplegia patient whose right upper extremity was fixed in a sling. Instructions for this procedure were provided by a researcher as, “please do not encourage movement of your right lower extremity during transfer to the wheelchair” [13]. The subjects had no musculoskeletal or neurological impairments. All the participants provided written, informed consent, and the study was approved by the local ethics committee of the Faculty of Medicine, Tottori University (number 2292).

### 2.2. Instrumentation

Three-dimensional (3D) motion analysis (MyoMotion Analysis System; Noraxon USA Inc., Arizona, USA) consists of combined motion sensors, surface electromyography (EMG), and synchronized video recordings. Signals of the subjects from these systems were digitally recorded (200 Hz and 1500 Hz and 30 Hz, resp.). In addition, the HR of each subject was measured via a wireless chest-strap electrocardiogram (ECG) monitor (Dynascope; Fukuda Denshi Co., Ltd., Tokyo, Japan). The motion sensor used Inertial Measurement Units, which are widely recognized as a means to overcome the disadvantages of existing optical motion capture systems [14]. The device can measure various kinematic parameters, such as object orientation and velocity, using accelerometers, gyroscopes, and magnetometers. The system has a measurement accuracy of 0.4 degrees for static measurements and 1.2 degrees for dynamic measurements.

### 2.3. Device

**Figure 1:**
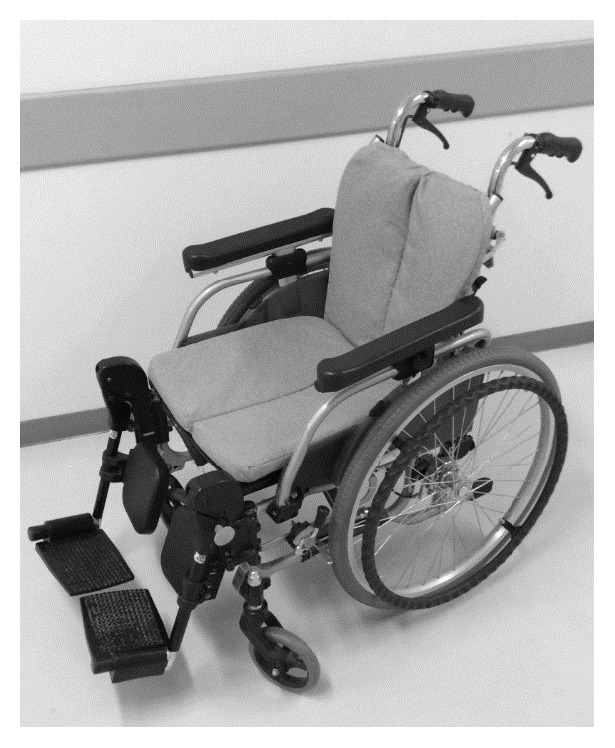
Conventional wheelchair (CWC).

**Figure 2:**
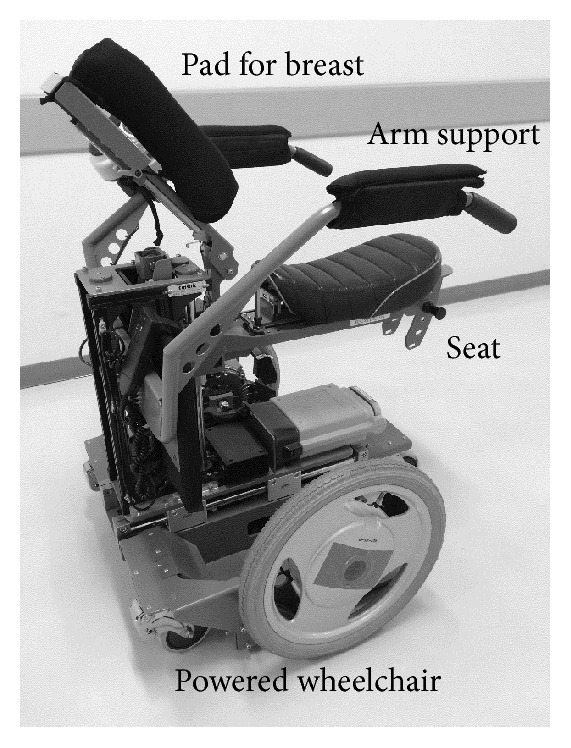
Robotic wheel chair (RWC). The seat in the RWC leans forward; thus, it makes the patients incline forward rather than lean back on the RWC. Patients are supported with a breast pad during sitting or driving. This device received a CE marking for safety and maneuverability. The criterion for using this device is the patients who have the ability to stand with assistance, and the exclusion criterion is the patients who have the inability to sit by oneself.

A standard CWC (Matsunaga, Co., Ltd., Gifu, Japan) was used in the study (Figure 1). The arm and foot supports could swing out upwards (seat width, 40 mm; front height, 42 cm; total length, 95.5 cm; weight, 18 kg). The RWC used in the study (Rodem, Tmsuk, Co., Ltd., Fukuoka, Japan) has an electric powered seat and a powered wheelchair. The seat moves back and forth and has an elevating mechanism to adjust the height of the patient (width, 720 mm; length, 750 mm; minimum turning radius, 360 mm; weight, approximately 80 kg; battery, lithium-ion battery) (Table 1 and Figure 2).

### 2.4. Experimental Procedure

**Figure 3:**
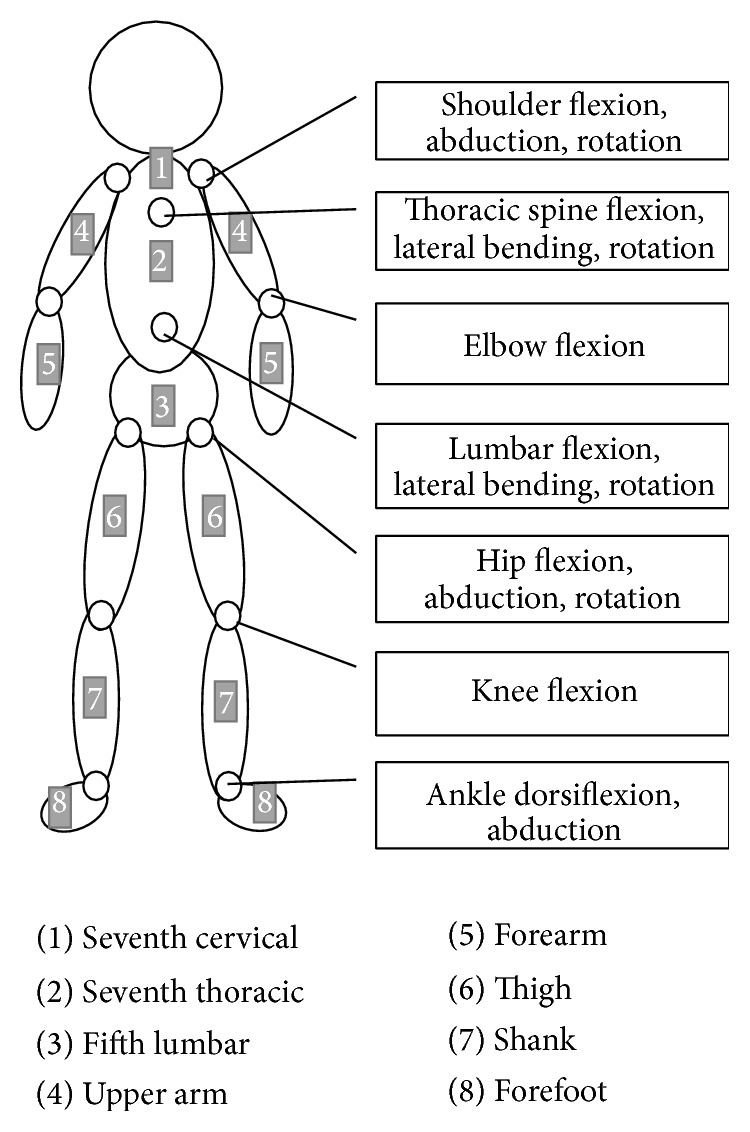
Placement of motion sensors and analysis of joint motion.

**Figure 4:**
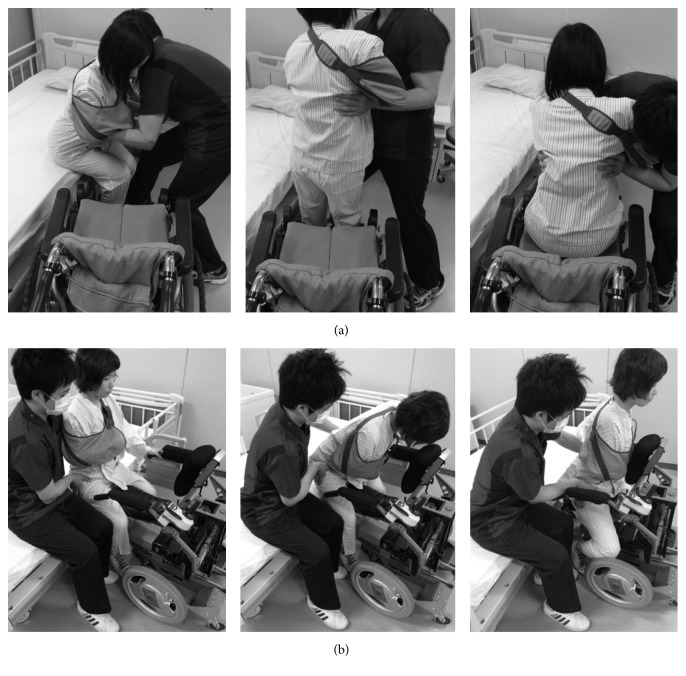
Transfer to the CWC and RWC. (a) Patient-handling tasks involved with a CWC can be classified into 3 groups: lifting of the patient, repositioning or turning from the bed towards the direction of the wheelchair, and seating the patient safely in the chair. (b) The RWC involves only 1 transfer step, which is that the assistant pushes the patient sitting on a bed forward in the same position to the seat of the RWC.

The motion sensors used for ROM measurements during transfer of the simulated patient were placed on the seventh cervical, seventh thoracic, and fifth lumbar vertebrae and bilaterally on the upper arm, forearm, thigh, shank, and forefoot (Figure 3). Calibration of the motion sensors was performed before the measurements using the segment model in the standing position. EMG electrodes were secured over the muscle bellies of both sides of the biceps, vastus medialis, upper back, and lower back muscles using standard techniques. Following practice, an EMG signal was recorded during maximum isometric manual testing of each muscle [15]. HR was assessed using a 3-electrode EKG (modified V5 lead). The signals from the motion sensors, EMG, and HR were recorded simultaneously in both experimental conditions (i.e., transfer using either the RWC or the CWC), while the subjects performed the following tasks, once in each situation and in random order: (1) CWC: the subjects supported the trunk of the simulated patient sitting on a bed and lifted the patient to a standing position, converted the patient's position toward the direction of the wheelchair, and seated the patient safely; (2) RWC: the subjects, located on the right side of the simulated patient who was sitting on a bed, supported the pelvis of the patient and pushed the patient directly onto the seat of the RWC. Transfer of the simulated patient was assisted until the patient was positioned on the seat (Figure 4). The RWC was located at the front of the patient with the height of the seat adjusted to the patient's sitting position using the robotic elevation system.

### 2.5. Data Analysis

Visual 3D motion analysis software (MR3; Noraxon USA Inc., Arizona, USA) was used to analyze the signal processing of the motion sensors, EMG, and video recordings. In both experimental conditions, the onset and cessation of ROM analysis, EMG, and ECG were defined as the start of assistance until the end of assistance to transfer the simulated patient and were determined by visual interpretation of the video recordings. ROM of the upper extremity, trunk, and lower extremity segments were calculated during both experimental conditions. In both situations, real-time ROM during patient transfer was analyzed using data obtained between 2 motion sensors; for example, right elbow-joint motion was analyzed using integrated signals of the accelerometers, gyroscopes, and magnetometers between the right upper arm and right forearm sensors, and the peak ROM was identified. For each muscle, the EMG data was integrated over 0.01 s intervals during each experimental condition and then normalized for each muscle's EMG signal of maximum voluntary contraction (MVC) which was recorded during maximum isometric manual test. Mean muscle activity, expressed as %MVC, and mean HR were calculated during both experimental conditions.

### 2.6. Statistical Analysis

All data were expressed as mean ± standard deviation (SD). Differences between the 2 experimental conditions were determined using paired *t*-tests. All statistical analyses were performed using SPSS for Windows Version 22 (IBM, Co., Ltd., Tokyo, Japan) and R ver. 3.0.3 (R Foundation for Statistical Computing, Vienna, Austria).

## 3. Results

### 3.1. Peak ROM on Motion Analysis

Table 2 shows the comparison of the motion analyses for CWC and RWC transfer. The peak ROM of both shoulder flexion and left ankle abduction during assistive transfer to the RWC were significantly lower than with the CWC. Left shoulder abduction, right shoulder rotation, and left knee flexion were significantly higher with the RWC than with the CWC.

### 3.2. %MVC on EMG Analysis

Table 3 shows the comparison of the muscle activation analysis for CWC and RWC transfer. The %MVC of the right biceps, the left upper back muscles, the left lower back muscles, and the right vastus medialis muscle were significantly lower with the RWC than with the CWC. The %MVC of the left biceps was significantly higher with the RWC than with the CWC.

### 3.3. ECG

There was a significant difference in mean HR during transfer between the 2 conditions (RWC, 87.1 ± 10.9 bpm versus CWC, 99.2 ± 13.2 bpm; *p* = 0.006).

### 3.4. Statistical Power Analysis for Sample Size

We performed power diagnoses for the tests with relevant outcomes and checked that most of them would have sufficient statistical power, for example, shoulder flexion (left 62.4%, right 67.6%), shoulder abduction (left 98.7%, right 8.5%), upper back muscles (left 99.2%, right 10.0%), lower back muscles (left 99.9%, right 20.5%), and HR (88.6%). Therefore, our statistician considers that the sample size was valid from a statistical perspective.

## 4. Discussion

Our study showed that transferring a simulated patient from bed to the RWC decreased the ROM on shoulder flexion, back muscle activity, and HR in the subjects compared to using a CWC. These findings suggest that the RWC may have the advantage of decreased muscle activity of the leg or back muscles during transfer compared with the CWC. This is because the RWC enables healthcare workers to push the patient forward in the sitting position; they may not have to lift the patients. This may decrease the musculoskeletal and physical strain on healthcare workers during transfer. In addition, transferring patients using a CWC involves 3 steps: lifting the patient, turning towards the direction of the wheelchair, and seating the patient safely in the chair. This adds complexity and requires the clinician to use more technical methods when transferring the patient. In contrast, the RWC does not involve lifting the patient, who instead can be transferred in 1 step using their own effort to push forward onto the seat of the RWC. It is simpler to assist in these movements. It may also be more comfortable for patients. During transfer to the RWC, the patient does not need to change direction while in the standing position as is the case with the CWC. Therefore, transferring to the front using the RWC may decrease the physical load on healthcare workers who often have to perform many patient transfers during a single working day.

The peak ROM values in the motion analysis of the subjects during transfer to the RWC showed lower shoulder flexion and left ankle abduction compared with transfer to the CWC. Transfer of patients from bed to the RWC does not involve lifting and also the foot position while sitting is neutral. In contrast, the technique for transfer to a CWC places higher demand on the shoulder flexion required to lift the patient, and a stride standing position and lower extremity abduction to maintain standing balance. It is not necessary to increase these ROM values when transferring a patient to a RWC. On the other hand, ROM of left shoulder abduction, right shoulder rotation, and right knee flexion were higher with the RWC. During transfer to the RWC, healthcare workers flex their knees to transfer the patient in the sitting position compared with a standing transfer. Shoulder abduction and rotation occur during transfer to the RWC because of the need to hold the pelvis of the patients using both arms. We suggest that the RWC has the benefit of possibly lowering ROM during transfers. However, pushing the patient forward in the RWC may place a burden on the upper extremities. It is therefore important to recognize the potential of shoulder abduction and rotation muscle overload during transfers using the RWC.

With the RWC, lower back muscle activity and lower mean HR during transfers were observed on EMG and ECG analyses. In the present study, analysis of the left back muscles during transfer from bed to the CWC showed 48%–66% MVC demand in the subjects. In a previous study, EMG analysis of the lower back muscles during transfer of patients to a CWC showed approximately 40% MVC in subjects [13]. We observed similar findings in our study, with the RWC decreasing left back muscle activity to a greater extent (i.e., %MVC of 29%–35%). It is also possible that HR increases because of increased muscle activity with a CWC compared to a RWC. In fact, a previous study showed that nurses rated patient lifting, transfer, and turning as the most physically demanding activities. Those tasks also correspond to the highest HRs recorded under clinical working conditions [4]. We suggest that this is an important advantage of the RWC compared to the CWC because of the need to decrease back muscle injury and cardiovascular stress during clinical work [4, 6]. Transfer from bed to the RWC requires healthcare workers to bend rather than rotate their trunks, without lifting the patients. This may decrease workplace injuries that occur during patient handling. This suggests that the RWC provides a simple transfer technique in which it is possible to transfer the load to the upper extremity when transferring patients with higher body weight.

The clinical implication of the RWC is that it represents a motorized device for older adults who are physically frail with weakness of the lower extremities and enables them to stand and turn to the wheelchair during transfer. In addition, we hypothesize that transfer using the RWC is advantageous for patients with Parkinsonism who cannot change their direction when standing. In this situation, we suggest that the RWC needs a robotic function or a sling to carry the patient in the sitting position to a seat. This would decrease the load on the upper extremities of the healthcare workers. We believe that riding the RWC is suitable for patients with pressure ulcers on the sacral area because the RWC does not have a back support; patients are supported by the breast support and do not have pressure on the sacrum. However, there is no available evidence regarding the pressure of the breast support or the seat. The weakness of the RWC is that this device does not have a back support, although the seat leans forward so that subjects do not fall backwards on the RWC. Thus, the use of this device is limited to those who have the ability to sit or stand with assistance. Additionally, transferring from the RWC to the CWC or bed is a weak point of this device because subjects who ride on the RWC must turn around and look behind in order to return to the bed. The RWC may need a rearview mirror or an automatic navigation system to correct this problem.

This study had several limitations. The patient was a simulated model. Therefore, the values obtained in our data analysis may not be applicable to the transfer of patients with paralysis, dysfunction of the lower extremities, or fractures. In addition, the sample size (*n* = 10) might not have been sufficiently large. However, we performed power diagnoses for the tests with relevant outcomes and checked that most of them would have sufficient statistical power. Also, as a result of the detailed experimental measurements that we made for all subjects, sufficient objective data for various indices and evidence that transfer of patients in the sitting positon clearly caused less physiological burden were obtained although repetitive load was not studied. The group of patients that would benefit most from transfers in the RWC and the associated implications are not addressed in the present study. Lastly, the study did not investigate muscle activity and psychological burden in the simulated patient. Future studies are therefore necessary to analyze these factors.

## 5. Conclusions

Shoulder flexion ROM, activity of the back muscles, and HR were decreased in the subjects when transferred from bed to the RWC. The RWC enables patients to be transferred in the sitting position directly to the frontal position. It is possible that the RWC will decrease workplace injuries and lower back pain in healthcare workers. Using an assisted device to transfer patients without manual lifting has obvious benefits in healthcare and rehabilitation settings.

## Figures and Tables

**Table 1:** Specifications of the robotic wheelchair (RWC).

Range of motion of seat	425 mm (up and down and/or front and back)
Speed of motion of seat	28 mm/s (up and down and/or front and back)
Speed	Advance: max. speed 4.5 km/h, min. speed 2.5 km/h
	Reverse: 2.0 km/h
Minimum turning radius	360 mm
Driving time	20 km
Weight	80 kg
Size	Length × width × height: 750 mm × 720 mm × 1,000 mm
Rear wheel size	16 inch
Number of wheels	Drive wheel × 2, training wheels × 3
Motor output	24 V 120 W × right and left side (AC motor)

**Table 2:** Peak ROM on motion analysis during transfer.

		CWC		RWC		95% CI			*p*
		Mean	SD	Mean	SD				
Lumbar flexion		35.6	12.7	35.8	12.6	−5.332	−	5.012	0.946

Lumbar lateral flexion		15.0	12.3	13.0	4.7	−7.508	−	11.646	0.637

Lumbar rotation		5.9	2.6	7.9	3.6	−5.960	−	1.920	0.276

Thoracic spine flexion		12.7	5.9	18.5	9.5	−12.824	−	1.282	0.097

Thoracic spine lateral flexion		13.7	7.4	13.7	3.4	−5.998	−	5.880	0.983

Thoracic spine rotation		12.6	5.4	13.0	5.2	−6.479	−	5.523	0.861

Elbow flexion	Left	61.9	28.8	57.7	14.3	−10.540	−	18.880	0.537
	Right	63.1	19.5	79.8	13.7	−38.339	−	4.899	0.114

Shoulder flexion	Left	72.7	16.1	57.1	12.7	1.780	−	29.380	0.031
	Right	65.9	11.1	52.4	12.4	2.254	−	24.866	0.024

Shoulder abduction	Left	24.3	11.5	48.3	12.1	−35.345	−	−12.503	0.001
	Right	35.6	18.2	30.7	12.8	−11.773	−	21.409	0.528

Shoulder rotation	Left	48.0	16.2	53.4	48.6	−38.067	−	27.267	0.717
	Right	34.5	14.3	63.0	16.7	−44.785	−	−12.135	0.003

Hip flexion	Left	71.6	10.4	83.9	19.9	−28.408	−	3.848	0.119
	Right	77.1	9.1	79.8	20.1	−17.129	−	11.629	0.675

Hip abduction	Left	28.5	10.4	19.8	15.4	−5.508	−	22.816	0.200
	Right	24.6	8.5	24.5	7.5	−7.859	−	7.999	0.985

Hip rotation	Left	29.8	11.5	22.9	13.5	−0.946	−	14.628	0.078
	Right	28.8	11.6	22.0	14.8	−9.111	−	22.663	0.360

Knee flexion	Left	63.2	9.1	97.1	7.5	−44.803	−	−22.977	0.000
	Right	71.0	15.5	83.9	29.4	−39.001	−	13.153	0.291

Ankle dorsiflexion	Left	20.1	7.7	20.0	12.3	−9.885	−	9.953	0.994
	Right	27.2	12.9	29.4	12.3	−16.824	−	12.306	0.734

Ankle abduction	Left	27.5	10.3	15.8	8.3	1.718	−	21.574	0.026
	Right	23.4	8.7	19.3	6.6	−4.326	−	12.386	0.304

ROM, range of motion; CWC, conventional wheel chair; RWC, robotic wheel chair.

**Table 3:** %MVC on EMG analysis during transfer.

	CWC		RWC		95% CI			*p*
	Mean	SD	Mean	SD				
Biceps muscle right side	65.6	31.6	34.9	29.8	13.167	−	48.179	0.003
Biceps muscle left side	62.7	37.0	90.1	44.0	−53.197	−	−1.603	0.040
Upper back muscles right side	59.6	25.5	66.9	33.4	−30.139	−	15.459	0.485
Upper back muscles left side	66.9	34.4	35.2	20.6	17.214	−	46.102	0.001
Lower back muscles right side	48.4	18.9	56.7	22.0	−22.898	−	6.438	0.236
Lower back muscles left side	53.9	25.0	29.4	18.9	16.699	−	32.331	0.000
Vastus medialis muscle right side	41.9	33.3	15.2	16.1	0.391	−	53.016	0.047
Vastus medialis muscle left side	53.0	50.2	37.5	48.4	−18.916	−	50.010	0.334

%MVC, % maximum voluntary contraction; CWC, conventional wheel chair; RWC, robotic wheel chair.

## References

[1] Blay N., Duffield C. M., Gallagher R., Roche M. (2014). A systematic review of time studies to assess the impact of patient transfers on nurse workload. *International Journal of Nursing Practice*.

[2] Feng C.-K., Chen M.-L., Mao I.-F. (2007). Prevalence of and risk factors for different measures of low back pain among female nursing aides in Taiwanese nursing homes. *BMC Musculoskeletal Disorders*.

[3] Kjellberg K., Lagerström M., Hagberg M. (2003). Work technique of nurses in patient transfer tasks and associations with personal factors. *Scandinavian Journal of Work, Environment and Health*.

[4] Hui L., Ng G. Y. F., Yeung S. S. M., Hui-Chan C. W. Y. (2001). Evaluation of physiological work demands and low back neuromuscular fatigue on nurses working in geriatric wards. *Applied Ergonomics*.

[5] Campo M., Weiser S., Koenig K. L., Nordin M. (2008). Work-related musculoskeletal disorders in physical therapists: a prospective cohort study with 1-year follow-up. *Physical Therapy*.

[6] Darragh A. R., Campo M., King P. (2012). Work-related activities associated with injury in occupational and physical therapists. *Work*.

[7] Black T. R., Shah S. M., Busch A. J., Metcalfe J., Lim H. J. (2011). Effect of transfer, lifting, and repositioning (TLR) injury prevention program on musculoskeletal injury among direct care workers. *Journal of Occupational and Environmental Hygiene*.

[8] Kjellberg K., Lagerström M., Hagberg M. (2004). Patient safety and comfort during transfers in relation to nurses' work technique. *Journal of Advanced Nursing*.

[9] Siddharthan K., Nelson A., Tiesman H., Chen F. F., Henriksen K., Battles J. B., Marks E. S., Lewin D. I. (2005). Advances in patient safety cost effectiveness of a multifaceted program for safe patient handling. *Advances in Patient Safety: From Research to Implementation*.

[10] Wang H., Tsai C.-Y., Jeannis H. (2014). Stability analysis of electrical powered wheelchair-mounted robotic-assisted transfer device. *Journal of Rehabilitation Research and Development*.

[11] Burnfield J. M., McCrory B., Shu Y., Buster T. W., Taylor A. P., Goldman A. J. (2013). Comparative kinematic and electromyographic assessment of clinician- and device-assisted sit-to-stand transfers in patients with stroke. *Physical Therapy*.

[12] Burnfield J. M., Shu Y., Buster T. W., Taylor A. P., McBride M. M., Krause M. E. (2012). Kinematic and electromyographic analyses of normal and device-assisted sit-to-stand transfers. *Gait and Posture*.

[13] Takasugi S., Ueshima T., Kusaba R. (2010). Robotic wheelchair device assist safety transfer and driving. *Clinical Rehabilitation*.

[14] Itami K., Fujita K., Yokoi K. (2004). A study of wheelchair transfer assistance using simulated patients trained to portray hemiplegic patients: patients safety, comfort and independence V.S. prevention of low back pain among nurses. *Ningen Kangogaku Kenkyu*.

[15] Christin H., Klaus B., Tim D. Ergonomic evaluation of upper limb movements in the automobile production measured by means of motion capturing.

